# Silver resistance in Gram-negative bacteria: a dissection of endogenous and exogenous mechanisms

**DOI:** 10.1093/jac/dku523

**Published:** 2015-01-06

**Authors:** Christopher P. Randall, Arya Gupta, Nicole Jackson, David Busse, Alex J. O'Neill

**Affiliations:** Antimicrobial Research Centre and School of Molecular and Cellular Biology, University of Leeds, Leeds LS2 9JT, UK

**Keywords:** efflux, Enterobacteriaceae, heavy metal resistance

## Abstract

**Objectives:**

To gain a more detailed understanding of endogenous (mutational) and exogenous (horizontally acquired) resistance to silver in Gram-negative pathogens, with an emphasis on clarifying the genetic bases for resistance.

**Methods:**

A suite of microbiological and molecular genetic techniques was employed to select and characterize endogenous and exogenous silver resistance in several Gram-negative species.

**Results:**

In *Escherichia coli*, endogenous resistance arose after 6 days of exposure to silver, a consequence of two point mutations that were both necessary and sufficient for the phenotype. These mutations, in *ompR* and *cusS*, respectively conferred loss of the OmpC/F porins and derepression of the CusCFBA efflux transporter, both phenotypic changes previously linked to reduced intracellular accumulation of silver. Exogenous resistance involved derepression of the SilCFBA efflux transporter as a consequence of mutation in *silS*, but was additionally contingent on expression of the periplasmic silver-sequestration protein SilE. Silver resistance could be selected at high frequency (>10^−9^) from Enterobacteriaceae lacking OmpC/F porins or harbouring the *sil* operon and both endogenous and exogenous resistance were associated with modest fitness costs *in vitro*.

**Conclusions:**

Both endogenous and exogenous silver resistance are dependent on the derepressed expression of closely related efflux transporters and are therefore mechanistically similar phenotypes. The ease with which silver resistance can become selected in some bacterial pathogens *in vitro* suggests that there would be benefit in improved surveillance for silver-resistant isolates in the clinic, along with greater control over use of silver-containing products, in order to best preserve the clinical utility of silver.

## Introduction

The silver cation (Ag^+^) has for centuries been employed as an antimicrobial agent. Current medical uses of silver include the prevention and treatment of bacterial infection in burns and chronic wounds;^1,2^ in 2009 alone, the UK National Health Service spent £25 million on silver-containing dressings for these purposes.^3^ In recent years, silver has also gained popularity outside of the clinic for its antimicrobial properties and is routinely incorporated into a variety of domestic and personal products, including paints, deodorants and clothing.^4,5^

The increasing use of silver for medical and non-medical applications has raised concerns that bacterial resistance to silver might proliferate in a manner analogous to that seen for antibiotics and thereby compromise its clinical utility.^6^ In a recent study to investigate potential silver resistance in the important Gram-positive pathogen *Staphylococcus aureus*, we found no evidence for extant resistance in a large collection of clinical staphylococcal isolates that included 876 strains of *S. aureus*, nor could any reduction in silver susceptibility be selected upon extended passage (42 days) of *S. aureus* in the presence of silver *in vitro*.^7^ Thus, it appears that the activity of silver is not under imminent threat from resistance in the staphylococci. However, growing silver usage could prompt a silver resistance problem in Gram-negative pathogens, particularly since silver resistance is already known to exist in several such species.^8–12^

**Figure 1.**
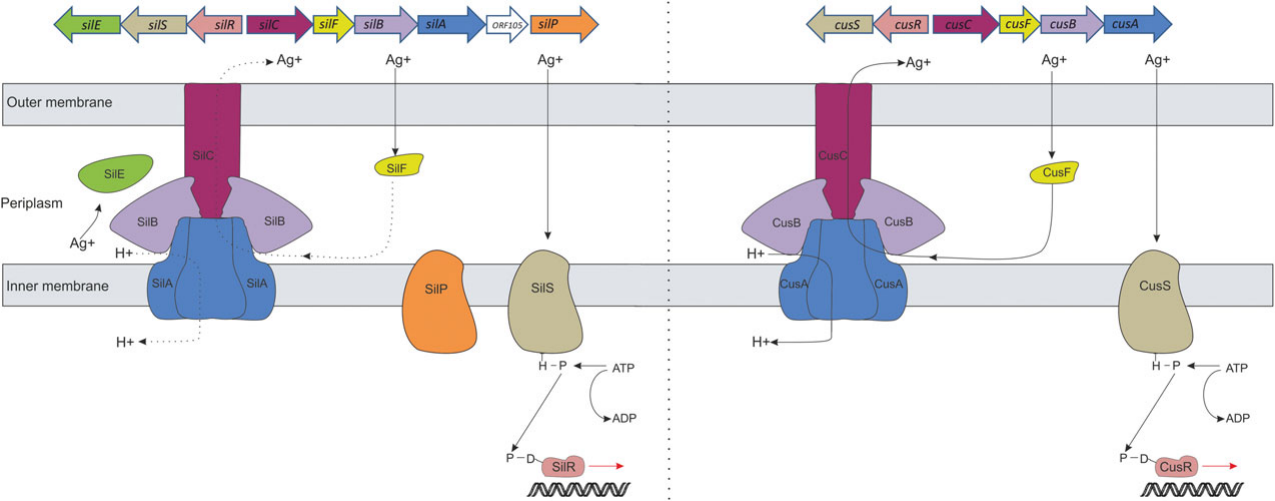
Comparison of the Sil and Cus systems and their proposed role in silver resistance. At the top of the figure, the genetic architecture of the *sil* and *cus* operons is shown; below that is a diagrammatic summary of what is known or predicted regarding the organization and function of these systems. The sensor kinase, CusS, responds to binding of Ag^+^ or Cu^+^ ions by phosphorylating the response regulator, CusR. Phosphorylated CusR mediates derepression of expression of CusCFBA. CusCBA is an RND-type Ag^+^/Cu^+^ efflux transporter that exports Ag^+^/Cu^+^ from the periplasm via an antiport mechanism, whereas CusF is a periplasmic Ag^+^/Cu^+^-binding protein that chaperones Ag^+^/Cu^+^ to CusCBA for subsequent efflux. Based on sequence identity, SilCFBA and SilRS are believed to have a similar function to their Cus counterparts. The Sil system includes some additional components not present in the Cus system: SilE is a periplasmic Ag^+^-binding protein, SilP is a hypothetical transporter of the P-type ATPase family and no putative function has been assigned to hypothetical protein ORF105. Dashed lines represent proposed interactions of Ag^+^/H^+^ with Sil system components.

Exogenous (horizontally acquired) silver resistance in Gram-negative bacteria was first encountered in a strain of *Salmonella* Typhimurium that caused an outbreak on a burns ward in 1975 and resulted in the death of three people.^8^ The genes responsible for silver resistance were subsequently mapped to a 14.2 kb region of the plasmid (pMG101) carried by this strain and have collectively been designated the *sil* operon.^13^ The *sil* operon comprises nine ORFs, seven of which are apparently structural genes (*silE*, *silC*, *silF*, *silB*, *silA*, *ORF105* and *silP*) and two of which (*silR* and *silS*) encode a putative two-component regulatory circuit (Figure 1).^13,14^ To date, only the function of SilE, a periplasmic silver-binding protein, has been determined.^13^ However, based on amino acid sequence similarity with other heavy metal resistance determinants (e.g. the Pco and Czc systems), the proteins encoded by the *sil* operon are believed to mediate silver resistance by restricting the accumulation of silver in the cell through a combination of silver sequestration in the periplasm (via SilE and SilF binding) and active efflux [via the resistance-nodulation-division (RND)-type efflux transporter SilCBA and the putative P-type ATPase transporter SilP],^13,14^ as detailed in Figure 1.

In addition to exogenous silver resistance, endogenous (mutational) resistance to silver has been reported in Gram-negative bacteria under laboratory conditions. Li *et al*.^12^ described the selection of five *Escherichia coli* mutants exhibiting ≥64-fold reductions in silver susceptibility compared with the parent strain. All five strains demonstrated loss of expression of one or more outer membrane porins (OmpF or OmpF/C), which apparently resulted in reduced outer membrane permeability, findings which imply that reduced silver susceptibility might in part result from restricted silver ingress into the cell. In addition, these strains were found to exhibit active efflux of silver out of the cell.^12^ Subsequent studies implicated CusCFBA (Figure 1) as the transporter responsible for silver efflux; components of the *cus* operon (CusF and CusB) were found to be overexpressed in an endogenously silver-resistant strain^15–17^ and deletion of *cusF* in this strain led to loss of the resistance phenotype.^15^

Whilst there is therefore some appreciation of the basis for both endogenous and exogenous silver resistance in Gram-negative bacteria, both systems remain incompletely characterized. As part of ongoing work to better understand the phenomenon of silver resistance in bacterial pathogens and its potential impact on the clinical utility of silver, we sought in the present study to gain a more detailed understanding of both types of Gram-negative silver resistance, with particular emphasis on clarifying their genetic bases.

## Methods

### General aspects

The bacterial strains and plasmids used in this study are described in Table 1. Bacteria were routinely cultured using Mueller–Hinton broth (MHB) and agar (Oxoid, Cambridge, UK), although LB broth and agar (Oxoid) were used to grow strains for genetic manipulation. Antimicrobial agents, including silver in the form of silver nitrate (AgNO_3_), and laboratory chemicals were from Sigma–Aldrich (Poole, UK).

**Table 1.** Bacterial strains and plasmids used in this study

Strain/plasmid	Comments	Reference/source
*E. coli* strains		
*E. coli* BW25113	derivative of *E. coli* K-12 strain BD792 (*lacI^q^ rrnB*_T14_ Δ*lacZ*_WJ16_ *hsdR514 ΔaraBAD*_AH33_ *ΔrhaBAD*_LD78_)	24
BW25113 *ΔompC*		24
BW25113 *ΔompF*		24
BW25113 *ΔompR*		24
BW25113 *ompR*_G596A_		this study
BW25113 *cusS*_G1130A_		this study
BW25113 *ompR*_G596A_ *cusS*_G1130A_		this study
BW25113 *ompR*_G596A_ *cusS*_G1130A_ Δ*cusC*		this study
BW25113 *ompR*_G596A_ *cusS*_G1130A_ Δ*cusF*		this study
BW25113 *ompR*_G596A_ *cusS*_G1130A_ Δ*cusB*		this study
BW25113 *ompR*_G596A_ *cusS*_G1130A_ Δ*cusA*		this study
*E. coli* J53 (pMG101)	silver-resistant *E. coli* K-12-J53 strain carrying plasmid pMG101	National Collection of Type Cultures
J53 (pMG101) Δ*blaZ*	as above, but with deletion of *blaZ* on pMG101	this study
J53 (pMG101) Δ*blaZ* Δ*silE*		this study
J53 (pMG101) Δ*blaZ* Δ*silC*		this study
J53 (pMG101) Δ*blaZ* Δ*silF*		this study
J53 (pMG101) Δ*blaZ* Δ*silB*		this study
J53 (pMG101) Δ*blaZ* Δ*silA*		this study
J53 (pMG101) Δ*blaZ* Δ*silG*		this study
J53 (pMG101) Δ*blaZ* Δ*silP*		this study
J53 (pMG101) Δ*blaZ* Δ*silGP*		this study
J53 (pMG101) Δ*blaZ* Δ*cusSRCFBA*		this study
J53 (pMG101) Δ*blaZ* Δ*cusSRCFBA ΔsilF*		this study
J53 (pMG101) Δ*blaZ* Δ*cusSRCFBA* Δ*silA*		this study
J53 (pMG101) Δ*blaZ* Δ*cusSRCFBA* Δ*silG*		this study
J53 (pMG101) Δ*blaZ* Δ*cusSRCFBA* Δ*silP*		this study
J53 (pMG101) Δ*blaZ* Δ*cusSRCFBA* Δ*silGP*		this study
*E. coli* DY441	source of *cat-sacB* counterselection marker	22
Other bacterial strains		
*E. cloacae* ATCC 13047		ATCC
*K. pneumoniae*	clinical isolate	Leeds General Infirmary
*S. sonnei*	clinical isolate	Leeds General Infirmary
*C. freundii* ATCC 8090		ATCC
Plasmids		
pKD4	source of FRT-*kan^R^*-FRT marker	40
pCP20	encodes FLP recombinase, ampicillin^R^, chloramphenicol^R^	40
pSIM6	encodes λ red recombineering system, ampicillin^R^	23
pSIM18	encodes λ red recombineering system, hygromycin^R^	23

### Susceptibility determinations and selection/phenotypic characterization of silver-resistant strains

MICs of AgNO_3_ were determined by serial dilution in MHB according to the CLSI broth microdilution method.^7,18^ The frequency of spontaneous mutation to silver resistance was assessed essentially as described previously.^19^ Passage experiments to select silver-resistant mutants were performed either by continuous exposure to subinhibitory concentrations of AgNO_3_ using the extended-gradient MIC method^7^ or by repeated exposure to supra-MIC concentrations of AgNO_3_, in a manner analogous to that described by Miller *et al.*^20^ The relative fitness of silver-resistant strains was determined by pairwise competition with the parent strain from which they were derived.^21^

### Whole-genome DNA sequence determination

A sample (1 μg) of total DNA from *E. coli* J53 (pMG101) was subjected to DNA sequence determination using the Illumina MiSeq platform at the Leeds Clinical Molecular Genetics Centre (St James' Hospital, University of Leeds). *De novo* assembly of sequence reads was performed using CLC Genomics Workbench, version 6 (CLC Bio, Cambridge, MA, USA) and annotated using the NCBI Prokaryotic Genomes Automatic Annotation Pipeline (PGAAP). The DNA sequence data for *E. coli* J53 (pMG101) have been deposited under GenBank accession number ASRI00000000.

### DNA manipulation

PCR was performed using Phusion polymerase (New England Biolabs, Hertfordshire, UK) in accordance with the manufacturer's instructions, and DNA sequence determination of PCR amplicons was performed by Beckman Coulter Genomics (Essex, UK).

Mutagenesis or deletion of genes was performed by recombination-mediated genetic engineering (recombineering) as described previously.^22^ Red recombination proteins were expressed from plasmids pSIM6 and pSIM18 in *E. coli* BW25113 and *E. coli* J53 (pMG101), respectively.^23^ For individual deletion of genes *cusC*, *cusF*, *cusB* and *cusA* in *E. coli*, the corresponding deletion mutants from the Keio collection^24^ were used as a source of the FRT-*kan*-FRT cassette. For all other gene deletions, plasmid pKD4 was used as the source of FRT-*kan*-FRT, which was amplified by PCR using the oligonucleotide primers listed in Table S1 (available as Supplementary data at *JAC* Online). Markerless deletions were subsequently generated by removal of the FRT-*kan*-FRT cassette from the site of integration by the FLP recombinase present on plasmid pCP20. Since pCP20 requires ampicillin selection for its maintenance, we used recombineering to first delete the native *blaZ* gene (conferring ampicillin resistance) from *E. coli* J53 (pMG101) prior to performing all other genetic manipulations. Allelic replacement mutagenesis of *cusS* and *ompR* was performed with *cat-sacB* counterselection.^22^

Expression of SilE in BW25113 *cusS*_G1130A_ was achieved from the *lac* promoter on plasmid pUC18. The *silE* gene was PCR amplified from *E. coli* J53 (pMG101) using oligonucleotide primers (Table S1) that contained engineered SacI (forward primer) and HindIII (reverse primer) restriction sites at their 5′ ends. The resulting amplicons were digested with SacI/HindIII, ligated into similarly digested pUC18 and introduced by transformation into *E. coli* DH5α, before recovery and transformation of BW25113 *cusS*_G1130A_.

### Analysis of cusC expression

RNA extraction, cDNA synthesis and quantitative (q) RT–PCR of *cusC* and 16S rRNA (internal control) were carried out as described previously,^25^ with the exception that first-strand cDNA synthesis was performed using the Omniscript RT kit (Qiagen) with oligo-dT(15) primers and RNase inhibitor (Promega) at final concentrations of 1 μM and 10 U, respectively. qRT–PCR was performed in an MxPro Mx3005P thermocycler (Agilent, Wokingham, UK) using the Quantifast SYBR Green PCR kit (Qiagen).

## Results

### Endogenous resistance to silver

#### Selection for endogenous resistance to silver

Attempts to select endogenous resistance to silver in *E. coli* strain BW25113 by plating saturated cultures onto agar containing silver at concentrations above the MIC (4 mg/L AgNO_3_) were unsuccessful (limit of detection <1 × 10^−10^). By contrast, endogenous resistance could be selected in BW25113 upon serial passage in the presence of sub-MIC concentrations of AgNO_3_ using the extended-gradient MIC method described previously.^7^ In three independent selections, a strain resistant to >256 mg/L AgNO_3_ arose on day 6 of the passage experiment. Attempts were also made to select resistance by repeated exposure of BW25113 to suprainhibitory concentrations of AgNO_3_ (40, 80 or 100 mg/L); however, after 42 days no reductions in silver susceptibility could be selected under these conditions.

#### Endogenous silver resistance results from two independent mutational events

Endogenous silver resistance in *E. coli* has been associated with loss of porins from the outer membrane and up-regulation of the native Cus efflux mechanism that is capable of transporting silver out of the cell.^12,15^ The genetic basis for these phenotypes is unknown and it has not been established whether they are necessary or sufficient to bring about resistance to silver. To identify the genetic changes that could underlie these phenotypes in our silver-resistant mutants, we subjected the following genes to PCR amplification and DNA sequencing: *ompF*, *ompC* and *ompA* (encoding the three major porins), *ompR* and *envZ* (encoding regulators of porin expression) and *cusR* and *cusS* (encoding regulators of expression of the CusCFBA efflux transporter). All three silver-resistant mutants harboured mutations in both *ompR* and *cusS*; all carried the same missense mutation G596A in *ompR* (encoding amino acid substitution R199H), but distinct missense mutations in *cusS* (T638G, C935A and G1130A, encoding I213S, A312E and R377H, respectively). To establish whether these mutations were both necessary and sufficient for the silver resistance phenotype, we used recombineering to reintroduce *ompR*_G596A_ and *cusS*_G1130A_, alone and in combination, into *E. coli* BW25113. Individually, neither mutation conferred an increase in the AgNO_3_ MIC compared with *E. coli* BW25113 (data not shown). However, when both mutations were introduced into the same strain, silver resistance (MIC >256 mg/L AgNO_3_) was observed.

#### Emergence and maintenance of endogenous silver resistance

**Figure 2.**
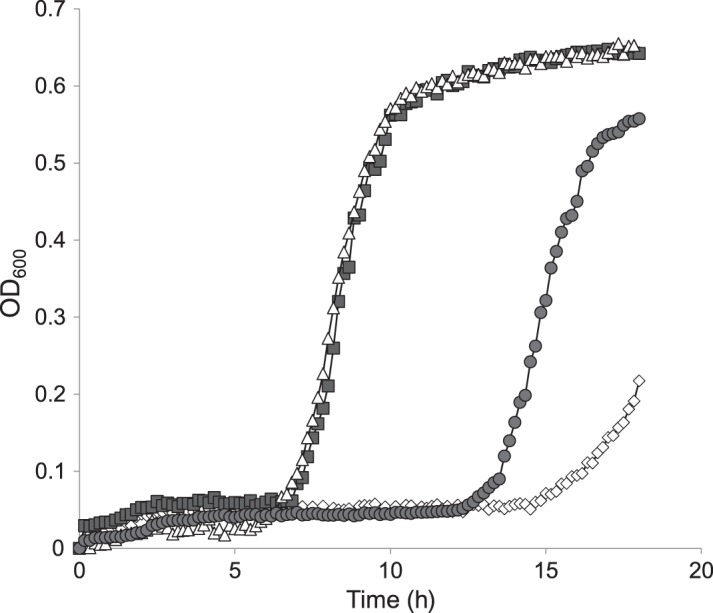
Growth of *E. coli* BW25113 and derivatives carrying mutations associated with endogenous silver resistance in the presence of 2 mg/L AgNO_3_. Open diamonds, BW25113; filled squares, BW25113 *ompR*_G596A_; open triangles, BW25113 Δ*ompR*; filled circles, BW25113 *cusS*_G1130A_.

We sought to establish which of the two mutations in our silver-resistant mutants had arisen first, an approach made possible by the fact that samples of cultures from each day of the passage experiment had been stored. PCR amplification and DNA sequence determination of the *ompR* and *cusS* genes from daily cultures recovered from all three independent passage experiments revealed that the mutation in *ompR* arose first after 4–5 days, whereas mutations in *cusS* arose on day 6. Although the *ompR* mutation did not affect gross silver susceptibility in standard MIC determinations, it presumably became selected because it nonetheless conferred a modest growth advantage over the parent strain BW25113 in the presence of silver. This was confirmed by evaluating the growth characteristics of BW25113 and BW25113 *ompR*_G596A_ in the presence of 2 mg/L AgNO_3_ (Figure 2). Under these culture conditions, a prolonged lag phase (15 h) was observed for BW25113, whereas BW25113 *ompR*_G596A_ entered the exponential phase of growth after only 7 h. We also assessed the ability of mutation in *cusS* associated with silver resistance (*cusS*_G1130A_) to confer a growth advantage in the presence of a sub-MIC concentration of AgNO_3_. Although a modest growth advantage was observed, it was less than that observed for strains carrying the silver resistance-associated mutation in *ompR* (Figure 2), thereby providing an explanation for the observed order in which the mutations in *ompR* and *cusS* arose under silver selection.

Experiments monitoring bacterial growth in the presence of a sub-MIC concentration of AgNO_3_ also revealed that BW25113 *ompR*_G596A_ and a strain deleted for *ompR* (BW25113 Δ*ompR*) demonstrated a comparable growth advantage over BW25113 (Figure 2), suggesting that the silver resistance-associated mutation in *ompR* causes loss of function of the OmpR protein. This was anticipated; OmpR is a transcriptional activator known to be required for expression of both OmpC and OmpF porins^26^ and only loss of function of this protein would be expected to reduce outer membrane permeability to silver by causing loss of porin expression. We confirmed through SDS–PAGE analysis of membrane fractions that neither OmpF nor OmpC was expressed in BW25113 *ompR*_G596A_ (data not shown).

Since genetic changes leading to loss of porin expression represent the initial event in the selection of endogenous silver resistance in *E. coli in vitro*, we reasoned that silver resistance would arise more rapidly in strains lacking porins prior to silver exposure, i.e. such strains would be primed for the development of endogenous silver resistance. Plating of saturated cultures of BW25113 Δ*ompR* (deficient in expression of both OmpC and OmpF) onto agar containing AgNO_3_ at 4× MIC recovered mutants exhibiting silver resistance (AgNO_3_ MIC >256 mg/L) at a frequency of 9.2 ± 1.2 × 10^−8^. The high frequency with which silver-resistant mutants arose is indicative of a single mutational event and PCR amplification and DNA sequencing of a selection of silver-resistant derivatives of BW25113 Δ*ompR* mapped the resistance mutation to *cusS* in all cases (data not shown). By contrast, silver resistance could not be selected under identical conditions using strains BW25113 Δ*ompF* and BW25113 Δ*ompC*, a result implying that both the OmpC and OmpF porins must be lacking to allow silver resistance to manifest. *E. coli* isolates lacking OmpC/F porins are extant in the clinic, often selected by exposure to β-lactam antibiotics.^27^ To ascertain if silver resistance can occur readily in such strains, we determined silver resistance mutation frequencies for *E. coli* EC18,^28^ a clinical isolate deficient in OmpC/F. A mutation frequency of 7.5 ± 0.9 × 10^−8^ was recorded at a selecting AgNO_3_ concentration of 4× MIC, with mutants resistant to >256 mg/L AgNO_3_.

**Figure 3.**
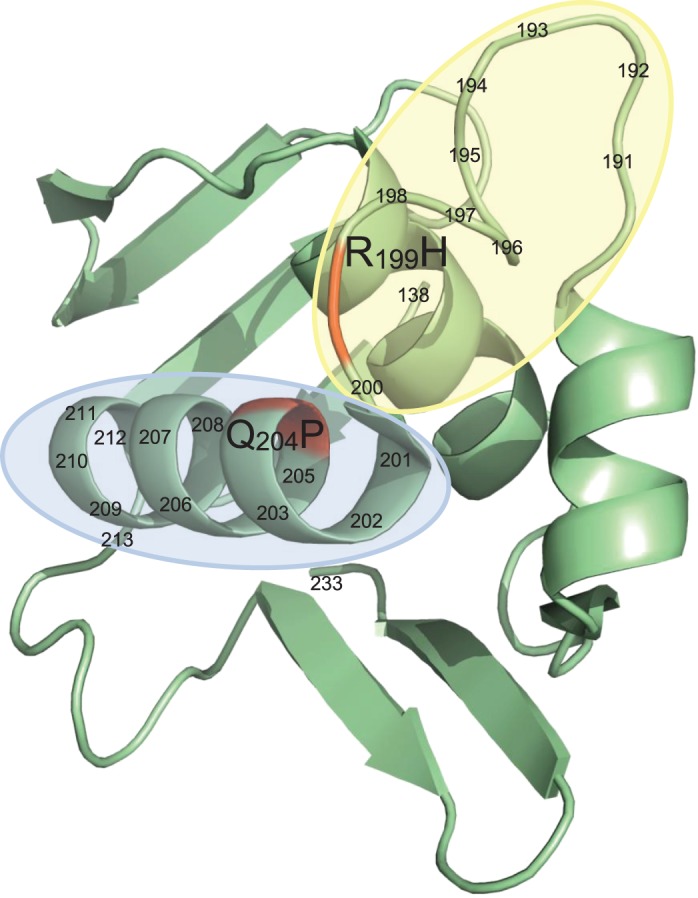
Amino acid substitutions in the OmpR protein that contribute to endogenous silver resistance in *E. coli*. Residues 138–233 of OmpR are shown, a portion of the protein that comprises a winged helix-turn-helix (PDB accession 10DD, originally presented by Kondo *et al.*^41^). The α3 helix of this domain (residues 200–13; highlighted blue) is responsible for DNA recognition and binding, whilst the ‘turn’ region (residues 191–99; highlighted yellow) within this domain recruits RNA polymerase to the site of OmpR–DNA interaction, allowing for transcription of genes including *ompC* and *ompF*.^41,42^ Amino acid substitution Q204P is located within the α3 helix, whilst R199H lies within the turn region; these substitutions likely mediate loss of function in OmpR by disrupting the ability of OmpR to bind DNA and to recruit RNA polymerase, respectively.

To provide an indication of the diversity of mutations within *cusS* and *ompR* that can participate in silver resistance, saturated cultures of strains *E. coli* BW25113 *cusS*_G1130A_ and BW25113 *ompR*_G596A_ were plated onto agar containing AgNO_3_ at 4× MIC. The DNA sequences of *ompR* and *cusS* were determined from at least four independent silver-resistant mutants arising from each strain. Mutations in *ompR* contributing to resistance included a nonsense mutation (C88T) and missense mutations encoding amino acid substitutions R199H and Q204P, both of which mapped within the C-terminal domains of OmpR (Figure 3). In the case of *cusS*, mutations conferring resistance led to amino acid substitutions that were not localized within any particular region of CusS, but were instead distributed along the length of the protein (T17P, I213S, A312E, A351E and R377H).

Endogenous silver resistance in *E. coli* has to date only been encountered under laboratory conditions; whether it occurs in the clinic is unknown. The observation that endogenous resistance can arise through two point mutations, or via a single point mutation in strains lacking OmpC/F porins, suggests that it could occur clinically under conditions of silver selection. To provide a further evaluation of the likelihood for endogenously silver-resistant *E. coli* to arise, survive and proliferate in the clinical environment, we determined the fitness cost associated with the silver resistance phenotype. Pairwise competition experiments with BW25113 and BW25113 *ompR*_G596A_ *cusS*_G1130A_ established a relative fitness value of 0.71 ± 0.02 for the latter, indicating a reduction in competitive fitness compared with the parent strain. However, since antibacterial resistance genotypes associated with greater reductions in *in vitro* competitive fitness than this have been isolated from patients,^29^ it seems unlikely that this degree of fitness impairment would represent a significant barrier to the emergence and spread of endogenous silver resistance in the clinical environment.

#### Silver resistance mutations in CusS derepress expression of CusCFBA, an efflux transporter that is essential for endogenous silver resistance

The CusRS two-component system is a positive regulator of the CusCFBA efflux transporter.^30^ Mutations in *cusS* participating in endogenous silver resistance presumably promote gain of function in CusS, which in turn leads to elevated expression of *cusCFBA* and ultimately to increased Ag^+^ efflux. To establish the effect of *cusS* mutations on expression of the *cusCFBA* operon, qRT–PCR was used to determine the relative expression of *cusC* in BW25113 and in the silver-resistant derivative BW25113 *ompR*_G596A_ *cusS*_G1130A_, in the absence of silver. The silver-resistant strain exhibited 6.2 ± 0.3-fold greater expression of the *cus* operon relative to the parental strain. Thus, mutations in *cusS* participating in silver resistance do result in gain of function and silver resistance is associated with elevated expression of *cusCFBA*.

Although CusF has previously been shown to be essential for endogenous silver resistance in *E. coli*,^15^ the essentiality of the CusCBA efflux transporter for silver resistance has not been established. To examine this, we independently deleted the genes encoding CusC, CusB and CusA in the silver-resistant strain BW25113 *ompR*_G596A_ *cusS*_G1130A_. Irrespective of the component deleted, the silver resistance phenotype was lost, with all three strains demonstrating an equivalent susceptibility to silver (4 mg/L) as the silver-susceptible parent strain BW25113.

#### Failure to select endogenous silver resistance in other Gram-negative genera harbouring the Cus system

The Cus system is not unique to *E. coli*. BLAST searching conducted during the present study revealed that it exists within a number of other medically important Gram-negative species, including *Citrobacter freundii* and *Shigella sonnei*. To determine whether endogenous silver resistance can also arise in these organisms, we subjected *C. freundii* ATCC 8090 and a clinical isolate of *S. sonnei* to continuous passage in the presence of sub-MIC concentrations of AgNO_3_, exactly as described above for *E. coli*. No reduction in silver susceptibility was detected in either strain after 42 days of continuous passage in the presence of silver, indicating that the presence of the *cus* operon in an organism's genome is not alone sufficient to ensure the emergence of endogenous silver resistance.

### Exogenous resistance to silver

#### DNA sequencing of E. coli J53 (pMG101) and sequence analysis of the sil operon

As mentioned above, plasmid pMG101 from *Salmonella* Typhimurium was the original source of the *sil* operon characterized by Gupta *et al*.^13^ This plasmid is a member of the IncH incompatibility group and in addition to silver resistance has been shown to confer resistance to ampicillin, chloramphenicol, mercury, streptomycin, sulphonamides and tetracycline.^8,31^ Although the nucleotide sequence of the region of plasmid pMG101 containing the *sil* operon has previously been determined (GenBank accession number AF067954), DNA sequence determination in our own laboratory of several *sil* operon components on pMG101 revealed some discrepancies. Consequently, we decided to resequence the *sil* operon and to generate, for the first time, DNA sequence information for the remainder of the pMG101 plasmid. To achieve this, we performed sequencing of total DNA recovered from *E. coli* strain J53 (pMG101). Following Illumina sequencing and *de novo* assembly, 113 contiguous sequences were obtained (with an N50 value of 103 568), of which 105 aligned to the previously deposited genome sequence of *E. coli* J53 lacking the pMG101 plasmid (GenBank accession number AICK00000000). The remaining eight contigs, comprising ∼151 kb of DNA sequence that did not align to the *E. coli* J53 sequence but that exhibited homology with other sequenced plasmids found in *Salmonella* species, represented plasmid pMG101. The DNA sequence of *E. coli* J53 (pMG101) has been deposited in GenBank under accession number ASRI00000000.

As described above, plasmid pMG101 confers resistance to a range of antimicrobial agents in addition to silver.^8^ The nucleotide sequence of pMG101 determined here revealed the basis for each of these resistance phenotypes: resistance to sulphonamides, mercury, streptomycin, ampicillin, chloramphenicol and tetracycline is mediated, respectively, by a sulphonamide-insusceptible FolP (locus tag L670_22583), the Mer system (L670_22518-22538), a streptomycin 3′-adenylyltransferase (L670_22593), a class D (OXA) β-lactamase (L670_22598), a chloramphenicol acetyltransferase (L670_22638) and TetA (L670_22816). Unexpectedly, the *sil* operon in *E. coli* J53 (pMG101) was found not to reside on pMG101, but had instead become integrated into the chromosome along with the copper resistance operon *pco*. Integration of the *sil* and *pco* operons into the chromosome from pMG101 appears to have been mediated by Tn*7*-based transposition, given the presence of Tn*7*-like elements in the integrant directly upstream of the *sil* operon. To calculate the approximate size of the original pMG101 plasmid with the *sil* and *pco* operons, the sizes of all eight contiguous sequences corresponding with pMG101 were added to the size of the integrated pMG101 fragment. This gave a total size for pMG101 of ∼183.5 kb, a figure close to the 180 kb estimate provided previously by Gupta *et al*.^13^

**Figure 4.**
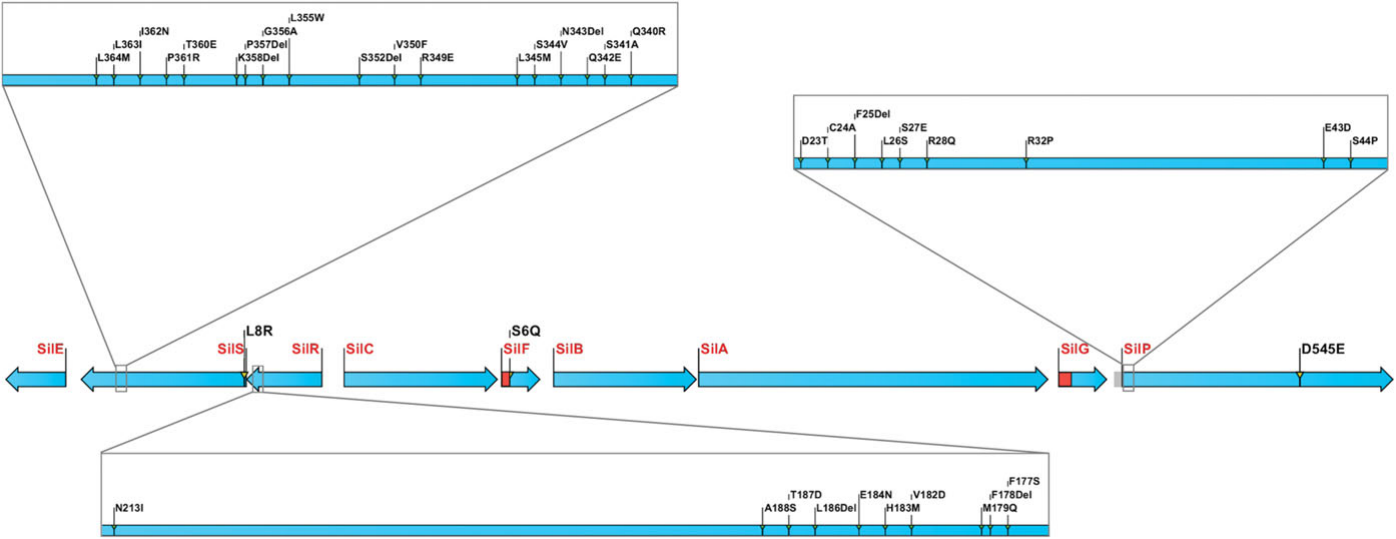
Resequencing of the *sil* operon reveals numerous coding differences compared with the previously published DNA sequence (GenBank accession number AF067954). Blue arrows represent the protein products of the *sil* operon. Coding differences are indicated, with the first letter in each case denoting the original amino acid and the second showing the change identified upon resequencing. PGAAP assignments of start codons for SilF, SilG and SilP are shown shaded red or grey for addition or deletion of amino acid residues, respectively. Del, deletion.

Comparison of the nucleotide sequence of the *sil* operon obtained in this study with the previously determined sequence (GenBank accession number AF067954) identified numerous discrepancies between the two. That these were not the result of DNA sequencing errors in the present study was confirmed by PCR and DNA sequencing of relevant regions of the *sil* operon from *E. coli* J53 (pMG101) (data not shown). In summary, 51 differences were identified between the two nucleotide sequences, comprising 10 base changes, 13 insertions and 28 deletions. Of these, 40 resulted in changes to the predicted amino acid sequence of Sil system components (Figure 4). In addition, the PGAAP algorithm used in this study to assign ORFs suggested start codons for *silF*, *silP* and *ORF105* different from those originally assigned by Gupta *et al*.^13^ and which, if correct, would alter the predicted amino acid length of the protein products encoded by these genes (SilF, from 96 to 117 amino acids; SilP, from 824 to 815 amino acids; and ORF105, from 105 to 146 amino acids). We have made the assumption in Figure 4 that the PGAAP assignment is more likely to be correct; however, experimental studies will ultimately be required to confirm these predictions.

**Figure 5.**
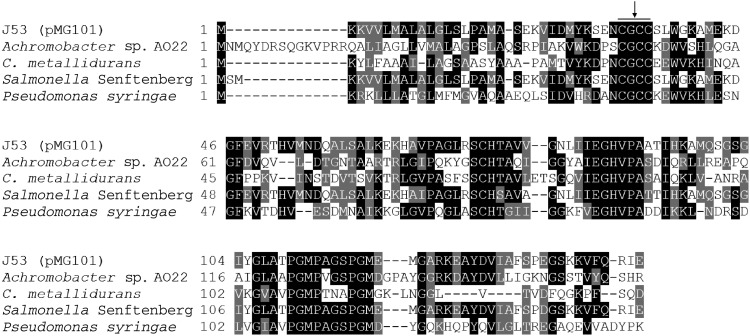
Alignment of the ORF105 protein from *E. coli* J53 (pMG101) with CopG proteins from various bacterial species. The conserved CxxC metal-binding motif is indicated. ORF105 has been redesignated SilG (please refer to the text).

The *sil* operon contains an ORF between *silA* and *silP*, designated *ORF105* in the original sequence submission, which appears to encode a protein for which not even a putative function has been assigned (Figure 1). BLAST searching with the encoded amino acid sequence of *ORF105* identified several homologous proteins in GenBank with sequence identities of ∼45%. These homologues included a putative copper chaperone termed CopG from *Cupriavidus metallidurans*^32^ and both CopG and ORF105 contain a conserved CxxC motif that is known to be involved in metal binding (Figure 5).^33^ ORF105 may therefore represent a metal chaperone. Bioinformatic analysis of this protein using the SignalP 4.0 server (http://www.cbs.dtu.dk/services/SignalP/)^34^ identified a putative N-terminal signal peptide, suggesting that, as for other components of the Sil system, this protein is localized in the periplasm (data not shown). Given the similarity of the gene product of *ORF105* to CopG, we propose that the former is renamed *silG* both to acknowledge this fact and to maintain the nomenclature of the *sil* operon.

#### Role of Sil system components in silver resistance

Whether all of the components encoded by the *sil* operon are required to mediate silver resistance has not been established. To address this question, we constructed a series of deletion mutants of *E. coli* J53 (pMG101) in which each of the *sil* genes was independently deleted and assessed the effect that this had on silver susceptibility (Table 2). Deletion of *silC*, *silB*, *silA* or *silE* led to complete loss of silver resistance, with the MIC of AgNO_3_ decreasing from >256 to 4–8 mg/L. By contrast, deletion of *silF*, *silP* or *silG* had no, or only limited, effect on the silver resistance phenotype (Table 2). Since the Sil and the Cus systems are homologous, and since *E. coli* J53 (pMG101) contains the *cus* operon as part of its genome, we considered the possibility that Cus system components could potentially act to complement for loss of SilF, SilP or SilG. By deleting the entire *cus* operon from *E. coli* J53 (pMG101), we found that this does appear to be the case for SilF; in the *cus*-negative background, deletion of *silF* resulted in complete loss of silver resistance (Table 2). By contrast, deletion of *silP* or *silG* in the *cus*-negative background, individually or in combination, had no detectable effect on the MIC of AgNO_3_ (Table 2).

**Table 2.** Silver susceptibility of *E. coli* J53 (pMG101) strains deleted for Sil and Cus system components

Strain	Silver nitrate MIC (mg/L)
J53 (pMG101)	>256
J53 (pMG101) Δ*blaZ*	>256
J53 (pMG101) Δ*blaZ* Δ*silE*	4
J53 (pMG101) Δ*blaZ* Δ*silC*	4
J53 (pMG101) Δ*blaZ* Δ*silF*	128
J53 (pMG101) Δ*blaZ* Δ*silB*	4
J53 (pMG101) Δ*blaZ* Δ*silA*	8
J53 (pMG101) Δ*blaZ* Δ*silG*	>256
J53 (pMG101) Δ*blaZ* Δ*silP*	>256
J53 (pMG101) Δ*blaZ* Δ*silGP*	>256
J53 (pMG101) Δ*blaZ* Δ*cusSRCFBA*	>256
J53 (pMG101) Δ*blaZ* Δ*cusSRCFBA ΔsilF*	8
J53 (pMG101) Δ*blaZ* Δ*cusSRCFBA* Δ*silG*	>256
J53 (pMG101) Δ*blaZ* Δ*cusSRCFBA* Δ*silP*	>256
J53 (pMG101) Δ*blaZ* Δ*cusSRCFBA* Δ*silGP*	>256

Despite the similarity of the Cus and Sil efflux transporters, only the latter system mediates silver resistance without the requirement for loss of porins from the outer membrane. Since SilE is the only protein of the Sil system essential for silver resistance that does not have a direct counterpart in the Cus system, it seemed probable that SilE acts to augment the action of the SilCFBA transporter, thereby obviating the need for porin loss. To provide confirmation for this, we examined whether SilE could also augment the action of the CusCFBA transporter, to bring about Cus-mediated silver resistance without porin loss. Introduction of pUC18:*silE* into BW25113 *cusS*_G1130A_ established that indeed it could, with the MIC of AgNO_3_ increasing from 4 to >256 mg/L.

#### Sil-based silver resistance in other bacterial genera

We established through BLAST searching that a number of other medically important Gram-negative species harbour the *sil* operon, including strains of *Enterobacter cloacae* and *Klebsiella pneumoniae*. To examine whether this operon also confers silver resistance in these species, the MICs of AgNO_3_ for *E. cloacae* ATCC 13047 and a clinical isolate of *K. pneumoniae* were determined. For both strains, AgNO_3_ had an MIC of 4 mg/L, a level of susceptibility equivalent to that seen for *E. coli* strains lacking the *sil* operon (i.e. fully silver susceptible). As established above for the *cus* operon, it may be that derepression of expression of the *sil* operon is required for silver resistance to manifest. Indeed, in a previous study, expression of the *sil* operon on pMG101 was shown to be constitutively high in comparison with that resident on other IncH plasmids that did not confer silver resistance.^31^ In an attempt to select spontaneous silver-resistant mutants of *E. cloacae* and *K. pneumoniae* in which expression of the *sil* operon had become derepressed, saturated cultures of these strains were plated onto agar containing AgNO_3_ at 4× MIC. For both strains, mutants exhibiting silver resistance (MIC >256 mg/L AgNO_3_) were recovered at frequencies suggestive of a single mutational event (1.9 ± 0.9 × 10^−8^ and 3.4 ± 1.7 × 10^−8^ for *E. cloacae* and *K. pneumoniae*, respectively). PCR amplification and DNA sequence determination of the *silS* gene from a single silver-resistant mutant of *E. cloacae* and *K. pneumoniae* identified mutations C638A (encoding amino acid change P213H) and G304A (encoding amino acid change A102T), respectively. Silver resistance mediated by the Sil system had minimal impact on bacterial fitness, resulting in a relative fitness of 0.91 ± 0.04 and 0.98 ± 0.02 for silver-resistant *E. cloacae* and *K. pneumoniae*, respectively, when compared with their silver-susceptible parental strains.

## Discussion

Our current understanding of the antibacterial mode of action of silver is that it is mediated predominantly through perturbation of the bacterial cytoplasmic membrane.^7^ In view of this, it is unsurprising that both endogenous and exogenous resistance to silver in Gram-negative bacteria involve mechanisms that act to restrict the accumulation of silver in the periplasm, since this will serve to limit the amount of silver reaching its antibacterial target. An essential component of both endogenous and exogenous resistance is silver efflux, mediated by the CusCFBA transporter in the case of endogenous resistance in *E. coli* and the SilCFBA transporter in exogenous resistance. These transporters are closely related homologues, with most of the counterpart proteins from the CusCFBA and the SilCFBA systems exhibiting amino acid sequence identities of >80% (data not shown). Furthermore, we have demonstrated here that SilE is able to augment the silver resistance phenotype associated with the CusCFBA transporter and that CusF is apparently able to complement for loss of SilF, findings that imply functional overlap between the two systems. The crystal structures of the CusCBA transporter and CusF have recently been solved^16,35,36^ and reveal that CusCBA utilizes a methionine shuttle to transport Ag^+^ and Cu^+^ out of the cell, whilst CusF utilizes cation–π and methionine interactions to bind a single Ag^+^ or Cu^+^ ion for delivery to CusCBA and subsequent efflux.^16,17,37,38^ In view of the high degree of primary sequence identity and the functional overlap between the Sil and Cus transporters, it seems highly likely that SilCFBA operates in the same manner. Thus, whilst endogenous and exogenous silver efflux in *E. coli* are genetically distinct phenomena, they apparently involve closely related mechanisms.

Expression of the Cus and Sil system efflux transporters is regulated in each case by a cognate two-component system (CusRS and SilRS, respectively) (Figure 1).^30,31^ In the absence of a stimulus (shown experimentally to be Cu^+^ or Ag^+^ ions for CusS and Ag^+^ for SilS), expression of these transporters is ordinarily repressed.^30,31^ Here, we have shown that in strains displaying a silver resistance phenotype involving either the Cus or the Sil system, derepression of transporter expression occurs owing to amino acid substitutions within the cognate sensor kinase (CusS or SilS). Although derepression of either CusCFBA or SilCFBA transporters is a requirement for silver resistance, the resulting increased efflux of silver is not alone sufficient to achieve an overt resistance phenotype. Presumably, the maximal rate at which these transporters are able to efflux silver is barely able to keep pace with the rate of silver ingress into the periplasm. Consequently, for an overt silver resistance phenotype to manifest, the effect of silver efflux must be augmented by mechanisms that act to restrict the accumulation of free silver ions in the periplasm.

In the case of endogenous resistance, this is achieved by restricting silver ingress as a consequence of loss of outer membrane porins. Although an earlier study using silver-resistant strains of *E. coli* selected *in vitro* suggested that loss of the OmpF porin was alone sufficient to restrict ingress to a level permitting Cus-mediated silver resistance,^12^ our present studies using defined deletion mutants have shown that OmpC and OmpF must both be absent. In agreement with this idea, we failed to recover endogenous silver-resistant mutants carrying mutations in individual porin-encoding genes. Instead, in all mutants selected, we observed simultaneous loss of OmpC/F porins as a consequence of mutation in *ompR*, the gene encoding the transcription factor responsible for activating OmpC/F expression.^26^ There has been extensive study of the structure and function of the OmpR protein, allowing us to make predictions as to how the different amino acid substitutions associated with silver resistance likely affect OmpR to prevent it from activating the expression of OmpC/F (Figure 3).

In contrast to endogenous silver resistance, exogenous resistance mediated by the Sil system is not contingent upon loss of outer membrane porins. Here, the effect of silver efflux is instead augmented by the action of the SilE protein, a concept supported by several lines of evidence. We have demonstrated that SilE is the only other Sil system component aside from SilCFBA that is essential for the exogenous silver resistance phenotype and there is no direct counterpart to this protein in the Cus system. Furthermore, engineering *E. coli* to carry both a derepressed *cusCFBA* operon and *silE* conferred silver resistance, without any requirement for loss of porins. In contrast to porin loss in endogenous resistance, which restricts silver ingress to the periplasm, SilE presumably has no effect on ingress and acts instead to reduce the periplasmic concentration of silver ions through sequestration.^13^ However, in the presence of high silver concentrations, the silver-binding capacity of the SilE protein would likely become exceeded, leading to silver concentrations in the periplasm reaching toxic levels. We therefore speculate that SilE does not only sequester silver ions, but also acts in a manner analogous to SilF/CusF and chaperones silver to SilCBA for efflux, either directly or via SilF (Figure 1).

The potential for selection of silver resistance in Gram-negative bacteria varies considerably between species. We failed to select any reduction in silver susceptibility in *Pseudomonas aeruginosa*,^7^ *Acinetobacter baumannii* (data not shown), *C. freundii* and *S. sonnei* during 42 days of continuous passage in the presence of AgNO_3_. By contrast, and under identical selecting conditions, endogenous resistance to silver (MIC of AgNO_3_ >256 mg/L) could be selected in *E. coli* in just 6 days. Furthermore, in some fully silver-susceptible Enterobacteriaceae, resistance could be selected in a single step (i.e. by a single point mutation) from (i) organisms harbouring a repressed *sil* operon (e.g. strains of *K. pneumoniae* and *E. cloacae*) or (ii) *E. coli* strains deficient in the OmpF/C porins. The frequency with which silver resistance can arise in some strains is therefore comparable to that (>10^−9^) seen for antibiotics acting upon a single cellular target, agents that are usually considered unsuitable for monotherapy owing to resistance liabilities.^39^ Furthermore, although both endogenous and exogenous mechanisms of silver resistance confer a fitness cost *in vitro*, this is unlikely to be sufficient to present a significant barrier to the emergence or spread of silver resistance in the clinic, particularly in settings where silver is in heavy use. The ease with which silver resistance can arise *in vitro* in some pathogenic Gram-negative species suggests that there would be benefit in surveillance programmes to monitor the emergence and spread of silver-resistant isolates, greater control over use of silver for medical applications and restricted use of antimicrobial silver for non-medical purposes. Collectively, these measures could serve to limit the development of resistance and thereby help to preserve the clinical utility of silver.

## Funding

This work was supported by an MRC DTG-CASE studentship with Smith & Nephew.

## Transparency declarations

C. P. R. has received travel grants from Smith & Nephew to attend international scientific meetings. All other authors: none to declare.

## Supplementary data

Table S1 is available as Supplementary data at *JAC* Online (http://jac.oxfordjournals.org/).

Supplementary Data
