# Supplementary material for: Silver resistance in Gram-negative bacteria: a dissection of endogenous and exogenous mechanisms
Source: J Antimicrob Chemother. 2015 Jan 6;70(4):1037–46. doi: 10.1093/jac/dku523 (PMC4356207; doi:10.1093/jac/dku523)
Supplement: Supplementary Data [file supp_70_4_1037__index.html]

Silver resistance in Gram-negative bacteria: a dissection of endogenous and exogenous mechanisms — Silver resistance in Gram-negative bacteria: a dissection of endogenous and exogenous mechanisms — Supplementary Data 

# Silver resistance in Gram-negative bacteria: a dissection of endogenous and exogenous mechanisms

## Supplementary Data

Supplementary Data

**Files in this Data Supplement:**

- Supplementary Data - Docx file
