# Supplementary material for: Silver resistance in Gram-negative bacteria: a dissection of endogenous and exogenous mechanisms
Source: J Antimicrob Chemother. 2015 Jan 6;70(4):1037–46. doi: 10.1093/jac/dku523 (PMC4356207; doi:10.1093/jac/dku523)
Supplement: Supplementary Data [file supp_dku523_dku523supp.docx]

**Supplementary data**

**Table S1.** Oligonucleotide primers used in this study

| **Name** | **Sequence** |
| --- | --- |
| **FRT-*kan*-FRT amplification primers** | |
| SilE ko forward | TTAGTGCAACTTATCAGGAAGGCTGGATTGCTTTATCAATATCCGGCGTCGTGTAGGCTGGAGCTGCTTC |
| SilE ko reverse | CGGAAAAATCTATCAAGGAAATAAGGACAGCACAGATACTTCCCTCGCCGCATATGAATATCCTCCTTAG |
| SilC ko forward | TCGGTAAAGTATCCCTATCAATACTCTGGACTTCGTTTGAACCATTTACCGTGTAGGCTGGAGCTGCTTC |
| SilC ko reverse | ACGGCTTTAAGTGAATTACGCATTTTAATTCCCTGATTAATTGAATAAATCATATGAATATCCTCCTTAG |
| SilB ko forward | TTACTGTGAATGAATGATCGTGCGCATATGCCAGGTGTTTTGATTTTTCAGTGTAGGCTGGAGCTGCTTC |
| SilB ko reverse | AACGGTTGGCGACAGAGCGCCGGATAATCCATTCAATCATCGTCGTCTCCCATATGAATATCCTCCTTAG |
| SilF ko forward | TGGGTGGCGGTTGGGTAGAGTAAATTTATTCAATTAATCAGGGAATTAAAGTGTAGGCTGGAGCTGCTTC |
| SilF ko reverse | CCATAATGAATGTTCAGGCGCACTGGATGTATTCGCTCCGGACTGTTAACATATGAATATCCTCCTTAG |
| SilA ko forward | TGTAAATATGCATTCAGGGCACTGAGGAGACGACGATGATTGAATGGATTGTGTAGGCTGGAGCTGCTTC |
| SilA ko reverse | GGGTGAATCAGTGCAGAAGGACGCCCACtGGGGGCGCCCTTTCAGGGTTAACATATGAATATCCTCCTTAG |
| SilG ko forward | CGTCCTTCTGCACTGATTCACCCTGACGTCAGGGTTTATATCGATAATATGTGTAGGCTGGAGCTGCTTC |
| SilG ko reverse | CGGCATAAGAGTGCCTGCTGCCGGCGTCCCGTTATCAGCCGTTCCGCTGACATATGAATATCCTCCTTAG |
| SilP ko forward | TGCATCCGGAGATACGCCGTAGTGGTCCCGGAAGCTGTCCTGTCTGTGGAGTGTAGGCTGGAGCTGCTTC |
| SilP ko reverse | TGCATCCGGAGATACGCCGTAGTGGTCCCGGAAGCTGTCCTGTCTGTGGACATATGAATATCCTCCTTAG |
| CusC ko forward | ACGAGATCGACCATCAAATC |
| CusC ko reverse | ATTAAACCTGGGTTACTGGC |
| CusF ko forward | ACCAGGCCAATCTGGATA |
| CusF ko reverse | AAATACTCACTCTGCGCTTC |
| CusB ko forward | TCTTTGTATACCGCACTTGG |
| CusB ko reverse | CAGTCCTGTAATGAGCGTAA |
| CusA ko forward | CGAAAATTCAGGGTATGGAC |
| CusA ko reverse | AATCCACGTTGGAACATCC |
| CusSRCFBA ko forward | GCGGCACGTTATTTTTACACTGGTTATAAAAGTTGCCGTTTGCTGAAGGAGTGTAGGCTGGAGCTGCTTC |
| CusSRCFBA ko reverse | GCCCATCGTCGCAAGACACAATCCACACGGTTAAACGGGGTATCCTGCTTCATATGAATATCCTCCTTAG |
| BlaZ ko forward | TGGCCGTCCCGACTTGATTGAAGGGTTGGGCGATTTTGCCATTAGATTTTGTGTAGGCTGGAGCTGCTTC |
| BlaZ ko reverse | GGGCGAACCCGGAGCCTCATTAATTGTTAGCCGTTAAAATTAAGCCCTTTCATATGAATATCCTCCTTAG |
| ***cat-sacB* cassette amplification primers** | |
| OmpR CS forward | TTTAAGAATACACGCTTACAAATTGTTGCGAACCTTTGGGAGTACAAACAATCAAAGGGAAAACTGTCCATAT |
| OmpR CS reverse | GAGCAATAACGTACGGGCAAATGAACTTCGTGGCGAGAAGCGCAATCGCCTGTGACGGAAGATCACTTCG |
| CusS CS forward | GAAGCTAATTCAGACCGTGCGCGGCGTGGGTTACATGCTTGAGGTGCCGGATCAAAGGGAAAACTGTCCATAT |
| CusS CS reverse | GCGGCACGTTATTTTTACACTGGTTATAAAAGTTGCCGTTTGCTGAAGGATGTGACGGAAGATCACTTCG |
| **Allelic replacement primers** | |
| OmpR forward | AGATTTAGCTGGTGACGAAC |
| OmpR reverse | ACCAGATAAGTCGTCACCAG |
| CusS forward | CGATACCAATGCTATTGATG |
| CusS reverse | ACCTTACATTAACGCTGGTT |
| ***silE* amplification primers** | |
| *silE* forward | TTCAGTATGAGCTCTCAGTGGACGCCAGGAAAT |
| *silE* reverse | ACCTTAATAAGCTTGCACAGATACTTCCCTCGCC |

Underlined nucleotides represent homology regions that allow for targeting of amplicons to the desired chromosomal location. Nucleotides highlighted in grey represent restriction sites.
